# A Case of Metastatic Choriocarcinoma With Intracerebral Hemorrhage Caused by a Ruptured Pseudoaneurysm

**DOI:** 10.7759/cureus.34522

**Published:** 2023-02-01

**Authors:** Hiroshi Kondo, Yoshihiro Kiura, Takuro Magaki, Tetsuhiko Sakoguchi, Atsushi Tominaga

**Affiliations:** 1 Neurosurgery and Neuroendovascular Therapy, Hiroshima Prefectural Hospital, Hiroshima, JPN

**Keywords:** ruptured pseudoaneurysm, metastatic choriocarcinoma, pseudoaneurysm, intracerebral hemorrhage, intracerebral metastasis, choriocarcinoma, uncommon stroke

## Abstract

A rare cause of cerebral hemorrhage is the metastasis of choriocarcinoma from gynecology. Herein, we report a case of a patient with brain metastasis of choriocarcinoma with cerebral hemorrhage. A 14-year-old female who had undergone surgery for a hydatidiform molar pregnancy presented with a disturbance of consciousness due to cerebral hemorrhage. Imaging studies revealed the presence of a cerebral aneurysm and several mass lesions in the lung field, and high serum beta-human chorionic gonadotropin level was confirmed. Thus, we suspected cerebral hemorrhage caused by brain metastasis of choriocarcinoma. She went into a coma, and an emergency craniotomy was performed to remove the hematoma and aneurysm. The pathology of the aneurysm was pseudoaneurysm due to the rupture of the vascular wall caused by increasing metastatic cells from choriocarcinoma in the cerebrovascular wall. Therefore, multidrug chemotherapy was immediately initiated. The choriocarcinoma, including the metastatic lesions, is in remission. To improve the outcome of choriocarcinoma, it must be diagnosed early, and treatment should be immediately started. Moreover, neurosurgeons should be aware of such diseases and consider them as one of the differential diagnoses, particularly in females of reproductive age with cerebral hemorrhage.

## Introduction

In females, genital choriocarcinoma rarely occurs after pregnancy. Such lesions are highly malignant, and distant metastasis occurs during the early period of their course [1]. Among all strokes, the rate of cerebral hemorrhage due to brain metastasis of choriocarcinoma from the gynecology field is rare. However, the rate of brain metastasis of choriocarcinoma is relatively high [2-4]. Because of its vascularity, there have been some reports of cerebral hemorrhage and aneurysm formation related to brain metastasis of choriocarcinoma [5-10]. Furthermore, when rebleeding occurs, the outcome is even worse [10], and early diagnosis and treatment are extremely important to achieve a good outcome. Therefore, neurosurgeons should be aware that brain metastasis of choriocarcinoma causes cerebral hemorrhage in females of reproductive age. Herein, we report a case of a patient with brain metastasis of choriocarcinoma. The intracranial hemorrhage was surgically evacuated, and the pseudoaneurysm was removed. A good outcome was achieved with early chemotherapy.

## Case presentation

**Figure 1:**
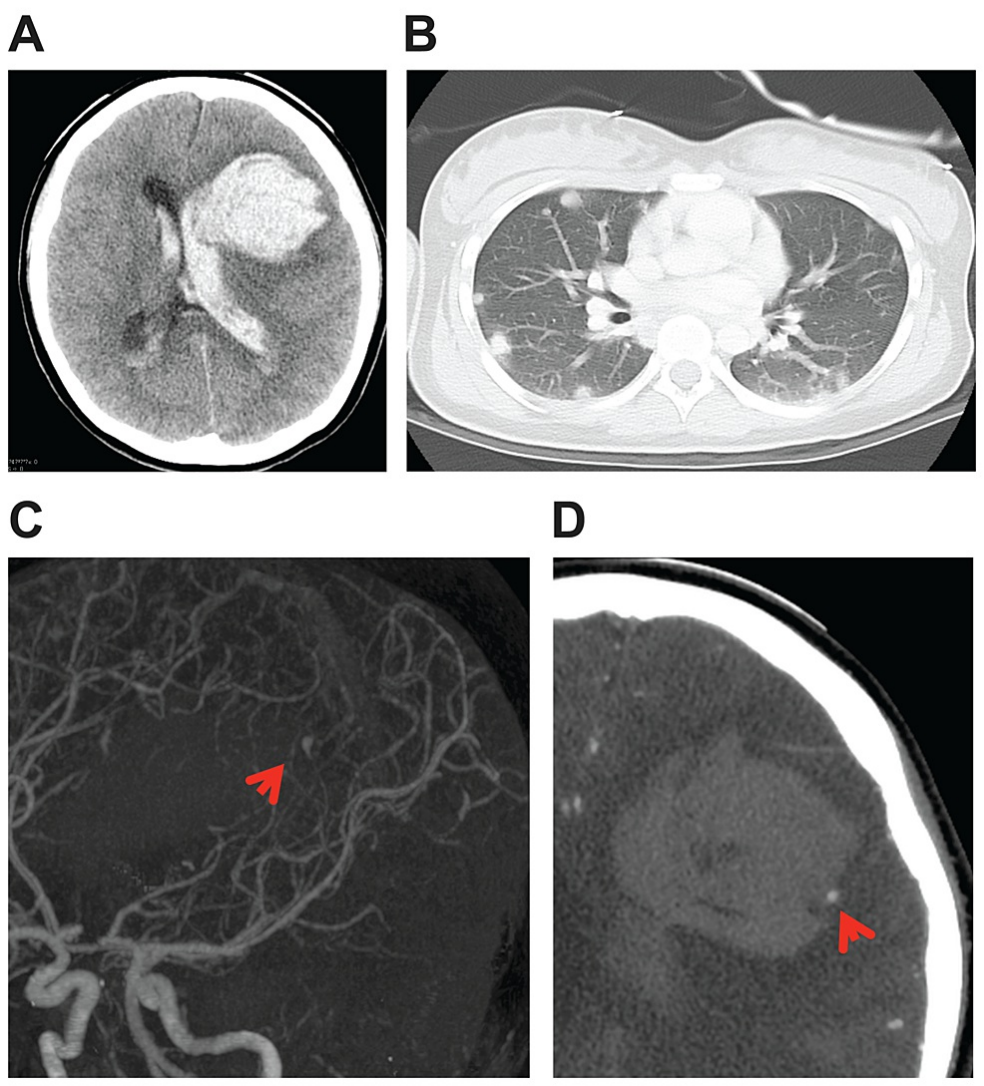
Images obtained in our hospital on admission (A) Head CT scan showed cerebral hemorrhage with ventricular puncture. (B) Chest CT scan showed multiple mass lesions in the lung field. (C, D) Three-dimensional computed tomography angiography revealed the presence of an abnormal vessel, which is suspected as an aneurysm, around the hematoma (arrow).

A 14-year-old female had undergone two surgeries for a hydatidiform molar pregnancy at a nearby hospital. Pathological diagnosis revealed a complete hydatidiform mole with no malignancy. The patient’s serous β-human chorionic gonadotropin (β-HCG) level was monitored, and it had decreased from 1,250,000 mIU/mL before surgery to 4016 mIU/mL 10 days after surgery. Two weeks after surgery, she presented with a disturbance of consciousness and was emergently transferred to a nearby hospital. A brain computed tomography (CT) scan revealed a subcortical hemorrhage in her frontal lobe with ventricular rupture (Figure 1A). She was then transferred to our hospital. The patient was in a coma and different pupils were confirmed. In our hospital, a chest CT scan revealed multiple mass lesions in the lung field (Figure 1B). In addition, three-dimensional computed tomography angiography (3D-CTA) revealed the presence of a hematoma, and an aneurysm or arteriovenous malformation was suspected (Figures 1C, 1D).

**Figure 2:**
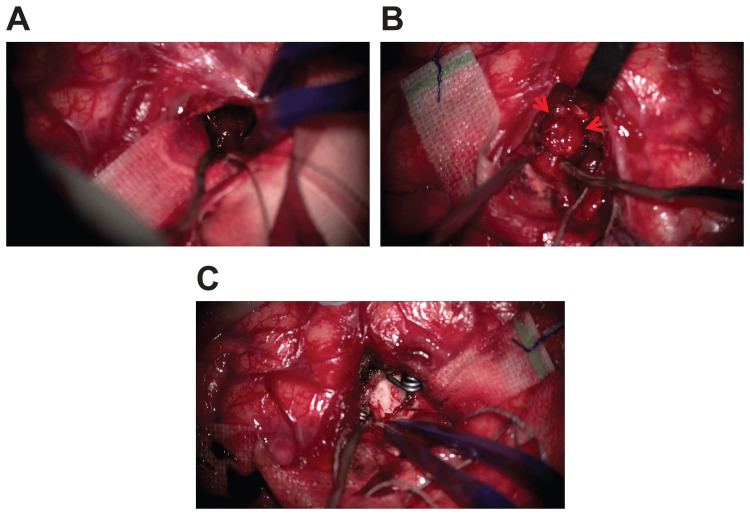
Intraoperative findings After creating a small corticotomy at the left frontal lobe and aspirating the subcortical cerebral hematoma (A), a pseudoaneurysm was observed in the hematoma (B) (arrow). The pseudoaneurysm was removed after the parent artery trapping (C).

Her serum β-HCG level was still high at 3181 mIU/mL. Thus, we suspected cerebral hemorrhage from a brain metastasis of choriocarcinoma. Emergency aspiration of the hematoma was performed. As the hematoma was aspirated, a pseudoaneurysm was found in the location where the abnormal vessel was found on 3D-CTA. Thus, it was resected after trapping (Figure 2).

**Figure 3:**
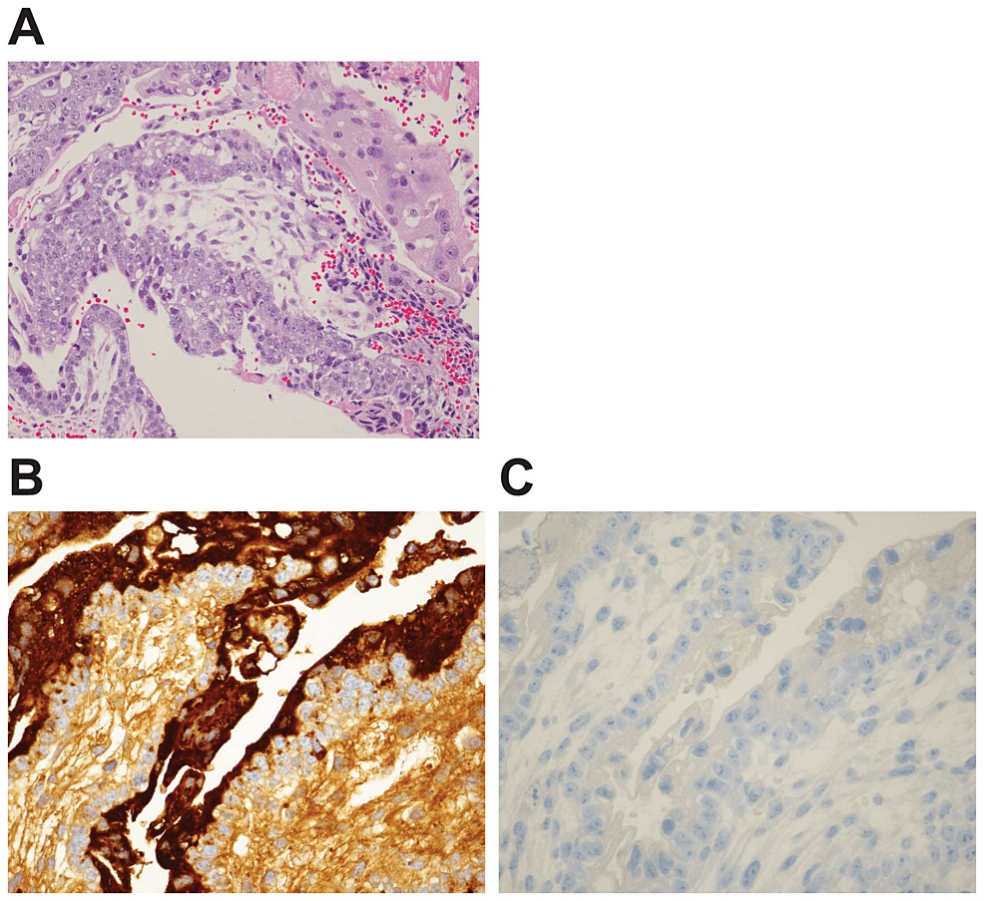
Pathological and immunohistochemistry findings (A) Hematoxylin and eosin staining findings: Syncytial trophoblast cells and cellular trophoblast cells with nuclear enlargement, but with almost no atypical mitotic figures, were found in the fresh hematoma in the pseudoaneurysm. (B) hCG staining findings: The syncytial trophoblast cells tested positive for hCG and PLAP. (C) PLAP staining: The cellular trophoblast cells tested negative for both syncytial trophoblast cells and cellular trophoblast cells. hCG: human chorionic gonadotropin; PLAP: placental alkaline phosphatase.

Based on pathological findings, syncytial trophoblast cells and cellular trophoblast cells with nuclear enlargement but with almost no atypical mitotic figures were found in the fresh hematoma in the pseudoaneurysm. Based on immunohistochemistry findings, the syncytial trophoblast cells tested positive for human chorionic gonadotropin (hCG) and negative for placental alkaline phosphatase (PLAP), and the cellular trophoblast cells tested negative for both hCG and PLAP (Figure 3).

**Figure 4:**
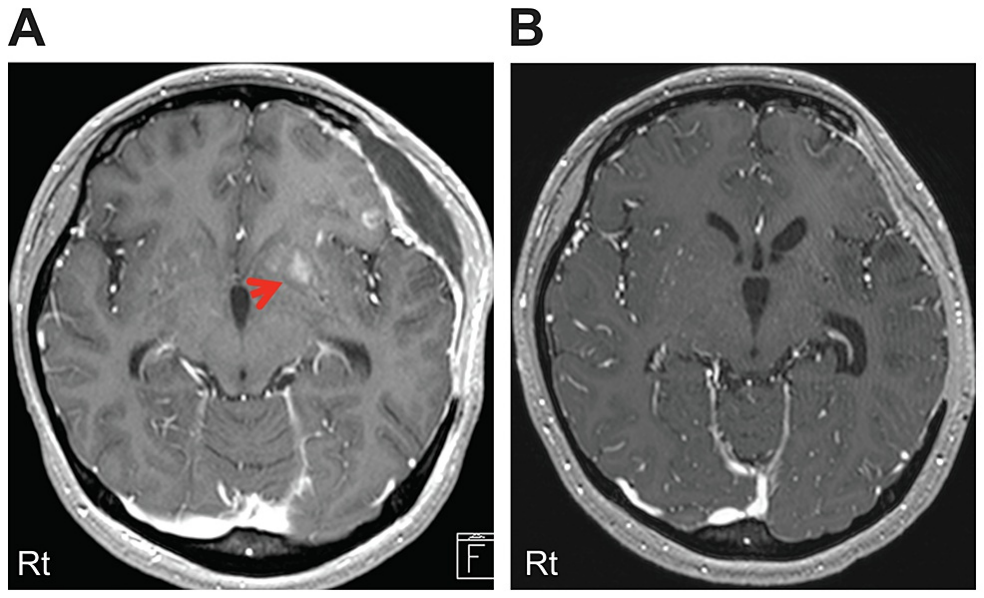
MRI findings (gadolinium enhancement) (A) Some gadolinium-enhanced lesions were observed in the frontal lobe before chemotherapy (arrow). (B) The gadolinium-enhanced lesions disappeared while the patient was on chemotherapy.

Similar findings were confirmed by CT-guided biopsy of multiple nodules in the lung field identified by chest CT scan. The patient was diagnosed with metastatic choriocarcinoma based on these data. In addition, she was classified under the high-risk group based on the International Federation of Gynecology and Obstetrics (FIGO) 2000 staging and the risk scoring system for trophoblastic neoplasia [11]. After surgery, her consciousness significantly improved. She then received seven courses of EMA-CO (etoposide, methotrexate, actinomycin D, cyclophosphamide, and vincristine) therapy and three courses of EMA (etoposide, methotrexate, and actinomycin D) therapy at the gynecology department. The contrast-enhanced lesions, which were observed by gadolinium magnetic resonance imaging (MRI) after surgery, disappeared while the patient was on chemotherapy (Figure 4).

The serum β-HCG levels decreased rapidly to below 0.1 mIU/mL, and the course of chemotherapy was completed. Neurologically, immediately after the operation, total aphasia and right hemiplegia were observed; thus, rehabilitation was continued. Although some higher brain dysfunctions were still observed, aphasia and right hemiplegia almost completely improved. Tumor recurrence was not observed for 24 months after the initiation of the treatment for the brain lesion.

## Discussion

Gestational trophoblastic neoplasias are caused by the abnormal proliferation of trophoblastic cells, and such conditions sometimes occur in young females after pregnancy. Among them, choriocarcinoma is a highly malignant lesion, and its high vascularity causes hematogenous metastasis in multiple organs at a high probability [1]. The incidence of brain metastasis is approximately 8.8-66.7% [2-4]. Some studies have reported the occurrence of intracranial hemorrhage [5-7] and subarachnoid hemorrhage after the formation of aneurysms due to the vascularity of the lesion [8-10]. Furthermore, when rebleeding occurs, the outcome is even poorer [10]. Therefore, early diagnosis and treatment are important to achieve a good outcome. In addition to clinical symptoms and imaging findings, a high serum β-HCG level is extremely important for diagnosis. In this case, cerebral hemorrhage due to the rupture of pseudoaneurysm occurred approximately two weeks after performing two uterine decongestions for hydatidiform pregnancy. Therefore, considering brain metastasis of choriocarcinoma, the measurement of serous β-HCG and whole-body imaging could be performed, and choriocarcinoma could be diagnosed early. As a result, after treatment for cerebral hemorrhage caused by the rupture of pseudoaneurysm, a patient could immediately receive chemotherapy. Choriocarcinoma and the metastatic lesions were in remission without any rebleeding, and a relatively good outcome could be obtained.

The first-line treatment for choriocarcinoma is EMA-CO therapy, and the remission rate of patients receiving such therapy is approximately 91% [12]. However, choriocarcinomas are highly vascular, and patients with such diseases are at risk of hemorrhage, which leads to neurological dysfunction and sudden death. Thus, brain metastasis is considered a risk factor for poor outcomes in patients with choriocarcinoma. Furthermore, Zairi et al. have summarized 20 previous case reports, including two of their own cases, in which a cerebral aneurysm developed due to metastasis of choriocarcinoma [9]. Among these patients, 13 died, and the outcome may more likely worsen when the aneurysms appear. A report has shown that an aneurysm caused by metastasis of choriocarcinoma has disappeared with chemotherapy [13], but these aneurysms are thought to easily rupture because an aneurysm caused by metastasis of choriocarcinoma is attributed to the destruction of the internal elastic lamina due to the growth of tumor cells in the blood vessel wall [14]. In the present case, pathological and immunohistochemistry findings confirmed the presence of a fresh hematoma and syncytial trophoblast cells and cellular trophoblast cells in the pseudoaneurysm; thus, the formation of an aneurysm due to the proliferation or tumor cells and its hemorrhagic property was observed.

EMA-CO therapy is effective and commonly used for choriocarcinoma. However, this multidrug chemotherapy is not effective in some cases. Xiao et al. have reported that the FAEV (fluorouracil, dactinomycin, etoposide, and vincristine) therapy combined with intrathecal injection of methotrexate was effective in patients with brain metastasis of choriocarcinoma who had a previous history of multidrug failure chemotherapy concomitant with renal metastasis or a FIGO score above 12 [4]. Whole-brain irradiation for brain metastasis has a certain effect in preventing bleeding and preventing the recurrence of brain metastasis. Although some studies have reported the use of radiation therapy in addition to multidrug chemotherapy, the remission rate is not very high [15-17]. By contrast, severe late neurotoxicity is frequently observed after whole-brain irradiation [18]. There has also been a report of the complete disappearance of intracranial metastatic lesions without radiation therapy [6]. In the present case, the ruptured aneurysm was removed by operation, and no other aneurysms have been found. Radiation therapy has a delayed effect, and treatment for preventing hemorrhage is not highly effective. Moreover, the patient was young, and considering the late neurotoxicity of radiation, only EMA-CO chemotherapy was continued after the operation without radiation therapy. The mass lesions in the brain and lung were completely distinguished, and the serum β-HCG level decreased to below 0.1 mIU/mL. The patient did not present with rebleeding, and no signs of recurrence were observed. However, some patients develop resistance to chemotherapy alone, and additional treatment such as intrathecal injection of methotrexate or radiation therapy should be considered.

## Conclusions

In females of reproductive age, brain metastasis of choriocarcinoma may be considered a cause of cerebral hemorrhage. To improve the outcome of such disease, early diagnosis and treatment are important; thus, the measurement of serum β-HCG level and whole-body imaging may be considered in female patients of reproductive age who present with cerebral hemorrhage. Neurosurgeons should remember that brain metastasis of choriocarcinoma is one of the differential diagnoses when treating cerebral hemorrhage in females of reproductive age.
